# Functional Effects In Silico Prediction for Androgen Receptor Ligand-Binding Domain Novel I836S Mutation

**DOI:** 10.3390/life11070659

**Published:** 2021-07-06

**Authors:** Alexey Rayevsky, Dmytro Sirokha, Dariia Samofalova, Dmytro Lozhko, Olexandra Gorodna, Inga Prokopenko, Liudmyla Livshits

**Affiliations:** 1 Laboratory of Bioinformatics and Structural Biology, Institute of Food Biotechnology and Genomics, National Academy of Sciences of Ukraine, 04123 Kyiv, Ukraine; o.v.raievskyi@imbg.org.ua (A.R.); samofalova.dariya@nas.gov.ua (D.S.); 2 Department of Molecular Modeling, Enamine Ltd., 02094 Kyiv, Ukraine; 3 Institute of Molecular Biology and Genetics, National Academy of Sciences of Ukraine, 03143 Kyiv, Ukraine; d.a.sirokha@edu.imbg.org.ua (D.S.); d.m.lozhko@imbg.org.ua (D.L.); algora@i.ua (O.G.); 4 R&D Department, Life Chemicals Inc., 1a DIXIE AVE, Niagara-on-the-Lake, ON L0S 1J0, Canada; 5 Department of Clinical & Experimental Medicine, School of Biosciences & Medicine, University of Surrey, Guildford GU2 7XH, UK; 6 UMR 8199-EGID, Institut Pasteur de Lille, CNRS, University of Lille, F-59000 Lille, France

**Keywords:** androgen receptor, ligand-binding domain, mutation, molecular modeling, molecular dynamics, time isolated RMSF, tunnel’s length and radius

## Abstract

Over 1000 mutations are described in the androgen receptor (AR) gene. Of those, about 600 were found in androgen insensitivity syndrome (AIS) patients, among which 400 mutations affect the ligand-binding domain (LBD) of the AR protein. Recently, we reported a novel missense mutation c.2507T>G I836S (ClinVarID: 974911) in a patient with complete AIS (CAIS) phenotype. In the present study, we applied a set of computational approaches for the structural analysis of the ligand-binding domains in a wild-type and mutant AR to evaluate the functional impact of the novel I836S mutation. We revealed that the novel I836S substitution leads to a shorter existence time of the ligand’s gating tunnel and internal cavity, occurring only in the presence of S836 phosphorylation. Additionally, the analysis of phosphorylation of the 836 mutant residues explained the negative impact on AR homodimerization, since monomer surface changes indirectly impacted the binding site. Our analyses provide evidence that I836S causes disruptions of AR protein functionality and development of CAIS clinical features in patients.

## 1. Introduction

Androgen insensitivity syndrome (AIS) is a genetic disorder of sex development (DSD) that occurs at a frequency of 1 in 20,000 live births, and is the most common DSD in people with karyotype 46,XY [1]. The AIS phenotypes range from normal female genitalia in patients with complete AIS (CAIS) to a wide range of ambiguous, undervirilized genitalia in patients with partial AIS (PAIS) [2]. Mutations in the androgen receptor (AR) gene are found in the majority of individuals with CAIS and in a small number of individuals with PAIS. The AR gene encodes for AR, which is a member of the nuclear receptor superfamily of ligand-dependent transcription factors. This nuclear receptor superfamily also includes estrogen, progesterone, mineralocorticoid, and glucocorticoid receptors. AR has the overall domain structure common to nuclear receptors, comprising an N-terminal activation domain (activation function 1 (AF1)), a central DNA-binding domain (DBD), and a C-terminal ligand-binding domain (LBD). Upon binding of ligand, steroid hormone receptors adopt an active conformation that facilitates the dissociation of heat shock proteins, dimerization, and binding to response elements in the promoters of responsive genes. During transactivation events, AR makes specific protein–protein interactions with several basal transcription factors, such as TBP (TATA box-binding protein) and TFIIF (transcription factor IIF). These interactions occur predominantly in the activation function-1 (AF1) region, located within a highly disordered N-terminal domain of AR [3]. A ligand-dependent interaction between the two AR termini is also necessary for maximum activation of the full-length receptor [4,5,6,7].

The main function of AR is a direct regulation of transcription. The binding of androgens to AR leads to a conformational change, allowing the transfer of receptor from cytoplasm to nucleus, where it functions as a homodimer [8]. Then, AR binds to a specific DNA sequence known as androgen response element (ARE) and interacts with other proteins in the nucleus to modulate transcriptional regulation [9]. The AR undergoes phosphorylation of certain amino acid residues which affect a variety of its functions, such as activation of the MAPK signaling cascade [10,11,12].

To date, more than 1000 mutations for AIS and prostate cancer are described in the AR gene [13]. Of those, about 600 were identified in AIS patients, with 400 mutations affecting the ligand-binding domain (LBD) of AR protein [13].

Our previous study in a Ukrainian cohort of patients with diverse AIS clinical phenotypes identified the novel missense mutation c.2507T>G (I836S, ClinVarID: 974911) in a patient with CAIS phenotype and affected family members [14]. This missense mutation is in exon 7 (ligand-binding domain, helix 9) and results in Ile836Ser substitution (Figure S1). This mutation is pathogenic, as determined by SIFT, PolyPhen, and MutationTaster [14].

We evaluated the effect of post-translational modifications (PTMs) on protein functionality, since PTMs increase structural and functional diversity of proteins. These PTM effects lead to a more complex impact on proteins' structure and function, as compared to the DNA backbone sequence. Therefore, it is crucial to uncover AR’s normal and pathogenic function. We determined the phosphorylation pattern changes and suggested that several known AR-specific kinases act upon mutant Ser836. These include PKC kinase (NetPhorest); MAPK family kinases (identified using Group-based Prediction System); and CDK1, CDK7, and CDK9 kinases from the Akt and MAPK families (identified using PhosphoPICK) [14,15,16,17].

In the current study, we investigated the CAIS phenotype-related impact of I836S mutation, and subsequent mutant variant phosphorylation, on AR functionality and on related pathogenic mechanisms. We combined approaches that predict and evaluate AR–LBD mutation pathogenicity. For this purpose, we conducted molecular dynamics (MD) simulations of wild-type and mutant monomers and dimers of AR–LBD, with and without dihydrotestosterone (DHT), in the binding site. We compared the obtained data for wild-type AR, established R832Q, Y835C, R841C, and novel I836S mutant (all located in helix 9).

## 2. Materials and Methods

All information about AR protein mutations that lead to AIS and their crystal structures were retrieved with the UniProt code P10275 (www.uniprot.org accessed on 16 January 2020). Crystal structures of AR (PDBID: 1R4I, 2AM9, 2PIQ, 5JJM, 4OEZ) in a high resolution were chosen from the RCSB protein database and represented the variety of conformations of the LBD [18]. Co-crystalized protein–ligand and protein–protein complexes (PDBID: 2PIQ, 5JJM, respectively) were used as the starting point for simulation of monomer and dimer. Both monomer and dimer structures were solvated with water model transferable intermolecular potential 3P (TIP3P) and a solute-box distance of 1.2 nm. All steps of MD simulations and analysis were performed using the Gromacs (2018.1, available at https://manual.gromacs.org/documentation/2018.1/index.html accessed on 03 February 2020) software tool and the CHARMM36 force field [19,20]. A constant system temperature during MD simulation experiments was maintained with a Nosé–Hoover thermostat. The simulations were performed at 310 K and 1 bar pressure, achieved with Parrinello–Rahman barostat. A cut-off radius of 14 Å was applied for both the Coulomb (electrostatic) and the Lennard–Jones (VdW) interactions using the particle-mesh Ewald (PME) method for calculation in the Verlet cut-off scheme [21]. DHT molecule was extracted from the crystal structure (PDBID: 2PIQ) and processed with a Swissparam.ch server to generate a topology compatible with the Gromacs environment [22]. To simulate a phosphorylated state of serine, we selected a topology of dianionic phosphoserine, which was substituted with PyMol 1.5 software (The PyMOL Molecular Graphics System, pymol.org accessed on 15 February 2020) [23]. We set the length of MD for monomers to 100 ns, and later extended it to 200 ns to confirm the accuracy of the obtained results. The dimer protein structures were simulated for a longer time, up to 500 ns, without extending the simulation. Radius of gyration (Rg), root mean square fluctuation (RMSF) and deviation (RMSD) analyses were carried out using Gromacs built-in tools. Based on the expected distribution of RMSD values in the first half of MD simulation, we analyzed a time-lapse corresponding to the second half, when the protein is already equilibrated and relaxed. Conformational consistency of the protein molecule was assessed with a built-in clustering module, which detected every leap of RMSD values, having a cut-off distance of 1Å for the two structures to be equivalent, and derived the conformational cluster’s number, distribution, and size of each cluster. The RMSF values were calculated for both the amino acid all-atom residues and the group of backbone atoms, with preference given to the latter [24,25]. The output RMSF indices characterized the motion of individual residues, while the higher frequency of convergences in the radius of gyration graph reflected better compactness of protein folding [26,27]. To obtain information about the overall stability and the changes in the amino acid mobility of the protein between the ligand-bound and unbound systems, we calculated the time-resolved RMSF of the ligand-free AR, as well as AR bound to DHT alongside the respective trajectories with the Visualizer Tool plug-in for VMD software package. This approach is more effective and convenient than generation and processing of multiple RMSD charts, reflecting a single residue’s motion at each moment of MD simulation.

As opposed to the standard surface generating software tools, which depict an external part of the protein, we applied an alternative analysis pack CaverAnalyst 2.0 (available at https://www.caver.cz/ accessed on 28 April 2020) for the cavity volatility assessment and explanation of distal mutation effect on the AR inactivation process [28]. The length of the tunnels and the radius were calculated for every snapshot of a trajectory, allowing the cavities inside the protein to be probed throughout the trajectory file. From these, a set of residues (Pro683, Leu708, Gln712, Arg753, and Phe765) were selected as a starting point for the tunnel search and volume calculations. To increase the accuracy of the search, we set approximation level to 15, minimum probe radius to 0.8, shell radius to 3 Å, and shell depth to 4 Å. Further clustering of the tunnels during MD simulations was performed by the average-link hierarchical Murtagh algorithm, with weighting coefficient of 1 and clustering threshold of 5.0 [29]. All mutations and PTM substitutions were prepared with PyMol, as well as visualization of structural changes, surface mapping, and pairwise alignments of trajectories from molecular dynamics simulations on final stages of the study.

## 3. Results

**Figure 1:**
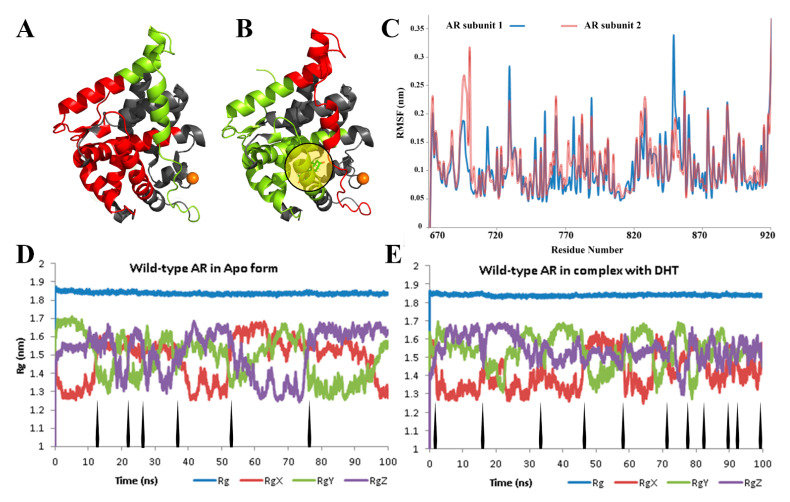
Stable structures of AR in ligand-unbound (**A**) and ligand-bound (**B**) states from the clustering of MD simulation. Color scheme: red—highly flexible residues, green—secondary structures with a decreased flexibility, grey—mobility does not depend on the binding state. DHT is indicated inside a yellow circle. The marked cartoon representation demonstrates the color change from green to red for residual mobility altering, and corresponding regions are numbered and specified on the RMSF plot (**C**). Despite increased flexibility (red color in B) of dimerization interface residues, the number of spikes/arrows on radius of gyration (Rg) graphs for DHT-free monomer (**D**) and DHT-bound structure (**E**) indicate higher stability of ligand-bound ternary structure. The calculated average radius of gyration includes three components Rgx, Rgy, and Rgz, which corresponds to the protein changes in x, y, and z directions.

We chose high-resolution ligand-bound wild-type protein structure (PDBID: 2PIQ) as an initial conformation for introduction of point mutations and all further simulations, upon inspection of all crystal structures available. Since I836S mutation is not directly related to protein–protein interaction (PPI) interface, we investigated the effect of ligand binding on a wild-type protein and determined the type of aforementioned structural changes that could be affected by the replacement of I836 residue. For this purpose, we conducted separate MD simulations of monomers and a dimer of AR-LBD with and without DHT in the binding site. The cleavage of the double bond between carbons 4 and 5 showed that DHT possessed the affinity for the AR at least twice as high, and five-times lower the rate of dissociation, compared to testosterone [30]. Figure 1 demonstrates the effect of the DHT binding on the stability of the wild-type monomer.

**Figure 2:**
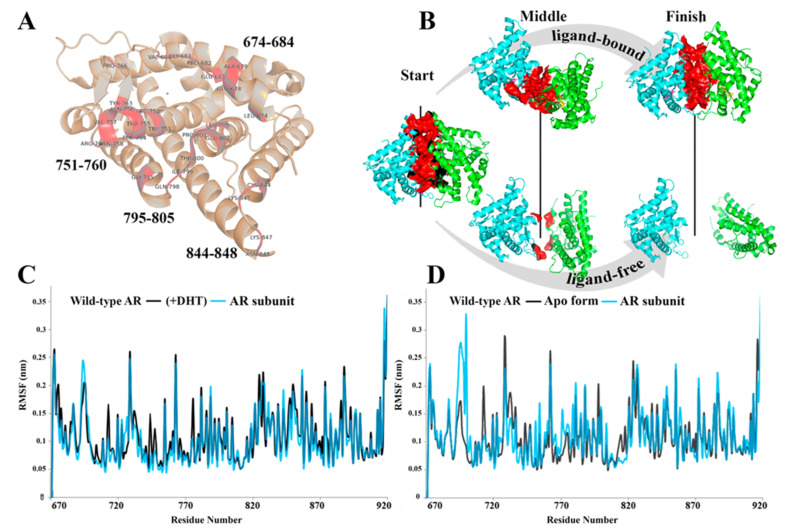
PPI interface forming residues of AR subunit (**A**). Dimer structure, colored by chain (cyan and green), with designated time-dependent protein interface (surface—colored in red) changes for ligand-bound and unbound AR dimer structures during MD simulations (**B**). Two comparative plots of RMSF for wild-type AR protein and subunit of the dimer demonstrate similar trends in residual flexibility response to DHT binding (**C**) or in the absence of DHT (**D**). The experimental data highlighted that ligand had stabilizing effect on LBD framework, the latter formed with ordered secondary structures. At the same time, a notable destabilization of multiple side chains and disordered loops of a single molecule of wild-type protein takes place (Figure 1A,B) [38]. These three loops are engaged in the PPI interface formation (Figure 2A). The homodimer structures behave differently in the absence and presence of the DHT molecule [8]. Furthermore, DHT-binding increases the molecular motions, which disrupt the LBD–LBD interaction formed in the cytoplasm (Figure 2B).

The binding of DHT might be responsible for conformational motion of N-terminal domain (NTD)–LBD of single AR molecules and their further translocation from cytoplasm to the nucleus [31,32,33,34]. Based on RMSD plots (Figure S2A), namely the plateau region, we concluded that the last 100 ns period of MD, following the initial 100 ns equilibration, is the most important period for analysis. Both the backbone-focused (Figure S2B) and sidechain focused RMSF graphs show increased flexibility of atoms in the ligand-bound complex. Thus, we determined the secondary structures, which altered flexibility of the ligand-bound conformation in the active site in response to the presence of a ligand molecule (Figure 2). The trajectory clustering revealed 110 and 41 different clusters for Apo and ligand-bound protein, respectively (Figure S2C,D). This type of structural analysis, along with radius of gyration values, demonstrated a significant rigidity of the ligand-bound conformation, in agreement with previous reports [35,36]. Additionally, analysis of the protein-ligand interaction throughout MD simulation was consistent with experimental data [37]. Specifically, the permanent and robust hydrogen bonds (H-bonds) are formed by Thr706 and Thr878, while the importance of H-bonds with Gln712 and Arg753 decreases continuously. These are ultimately replaced with contacts formed by Phe765 and Met746.

Our simulations of the separate LBD dimer during 500 ns resulted in an early dissociation of ligand-free monomers after 360 ns, while the ligand-bound complex remained in its original dimer form (Figure S3). The reason for the protein–protein complex disintegration lies in a partial violation of contact map. This deformation could be a consequence of the ligand binding and conformational shifts in LBD, which affect other domains of AR structure.

The analysis of both dimer complexes showed that activation of AR with DHT leads to an increased flexibility of two loops, which form PPI interface. For these loops, we analyzed the trajectories of dimeric AR complexes, containing either ligand-bound or ligand-unbound subunits. The comparison of the RMSF plots showed expected stabilization of the structure, where the RMSF graph is similar to trajectories of the ligand-bound AR monomer. The residues of PPI interface can retain some flexibility and thus contribute to PPI interaction (Figure S4 and Tables S1–S4). After disruption, the AR monomers may take part in N/C terminal interactions to provide its relocation in the nucleus [7]. The intra-nuclear dimerization of DNA binding could be facilitated, in part, by some transcriptional coregulators [39,40].

**Figure 3:**
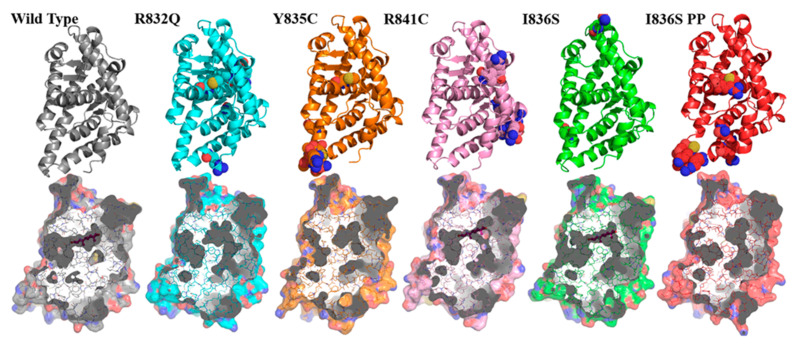
Stable conformations derived in MD simulations of the mutant proteins demonstrate some visually detected local flexibility of residual clusters (ribbon-like structures on the top) in various regions of protein structure (shown with colored dots). A surface-based representation (bottom) applied to show the collapse of the binding site tunnels, as a response to introduced mutations. The models, which preserved the open-gated mode, are shown with a DHT structure in the background (wild type, R841C, I836S).

As we mentioned above, the dimerization process may not take place, either because of DHT binding failure or due to PPI interface changes. The protein surface alterations, which could directly break the PPI or indirectly impact the ligand-binding residues, usually come from the large amplitude secondary structure shifts. There is no established molecular mechanism of disease development as a result of a mutation in helix 9. However, several mutations (R841C, R832Q, and Y835C) in this region are associated with CAIS development. These mutations were experimentally confirmed to reduce the ligand binding capability of AR [41,42,43]. Our previous sequence-based analysis suggests that serine residue, replacing isoleucine in the 836 position, can occasionally be phosphorylated (I836S pp). Hence, we conducted five simulations to shed the light on this sequence of events (Figure 3).

RMSF analysis of the ligand-free complex was used as a negative control for this set of analyses (Figure S5). Despite some growing flexibility, all structural changes were not as dramatic as those shown for the wild-type apo- and ligand-bound forms. Surprisingly, the results of the residual mobility graph analysis for DHT containing mutant were remarkably similar to those from the ligand-bound wild-type trajectory (Figure S5). Expecting an increased movement of S836 with a voluminous and charged phosphate group over the unmodified residue, we performed the same range of analysis. Based on the RMSF indices, calculated for individual residues, we found that PPI interfaces of all instances were almost identical. It is worth noting that DHT stabilizes mutant protein, based on gyration radius graphs (Figure S6). MD simulation showed that R841C mutation caused the moderate binding site closure.

Despite observed flexibility of individual residues, our analysis did not reflect any visible trend, which could explain the rearrangement of signal transduction across protein structure. The protein complex with androgen did not show the effect of mutation. LBD itself was represented with a tightly folded globule, but the activation function 2 (AF2) segment formed a highly ordered hydrophobic surface, structural integrity, and further activation level, which depends on androgen binding [44,45]. As our mutation hits the molten globule-like AF2 region, it makes no notable impact on the external surface changes, given sufficient space is available for free deformations. However, the volume of cavities is limited by the protein’s framework. Consequently, even a small shift in residual position in the binding site entrance or its bottom can affect the functionality of the entire protein. We realized that routine investigation of the RMSF, RMSD, or Rg data is not sufficient for complete understanding.

All mutations (I836S and R841C, R832Q, Y835C) caused the binding site closure and may lead to impossibility of the ligand entrance. It is vividly noticeable when the comparison with the Apo-form of wild-type protein is made.

**Figure 4:**
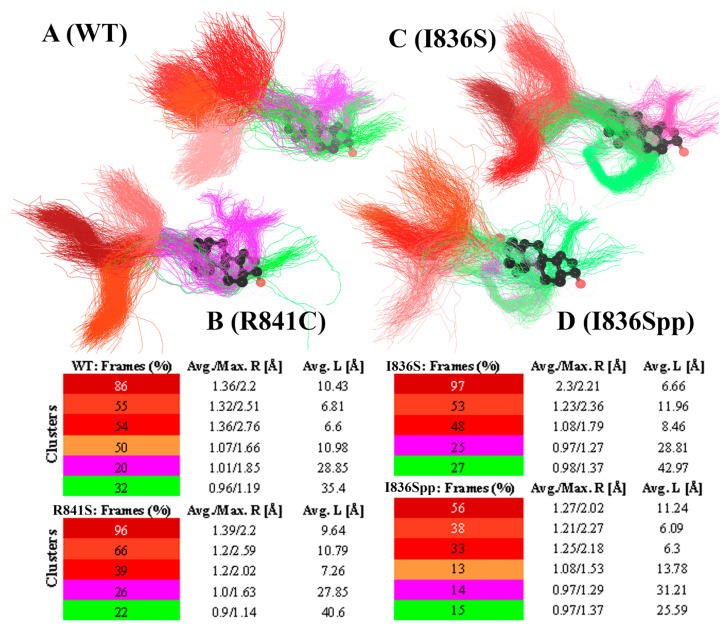
The trajectory analysis of a WT protein simulation (**A**), annotated R841C mutation (**B**), dephosphorylated and phosphorylated replacement of I836 with serine (**C** and **D**, respectively). Spatial alignment of observed binding site cavities (similar cavities from distinct objects are painted with the same color), based on centroid lines of tunnels, colored by corresponding tunnel cluster with imposed DHT structure. Corresponding tables contain the information about the time of each tunnel’s part existence/occurrence of each cluster separately in percent representation (if 100% is existence of a particular cluster/tunnel throughout the whole simulation) and spatial parameters, including average (Avg.) and maximum (Max.) tunnel radiuses (R) and average length (L) derived in MD simulation trajectory.

The trajectory analysis of mutant proteins, provided with a tunnel calculation module, showed that diameter of the gateway in the binding site gradually decreased, and then collapsed after 140 ns of MD for the mutant, while a similar site in a wild-type protein remained open. The collapse of the binding site was provoked by tension, causing H1/H2 helices offset and, finally, disposition of Val685, Glu682, Gln712, Met750, and Arg753 residues involved in forming the entrance of the binding site. Notably, phosphate substitution was directly relevant to the described mechanism, and unmodified residual replacement had no dramatic effect on the binding site shape. Figure 4 demonstrates visual alignment of tunnel centroid lines with additional data on the shape of tunnel clusters observed during MD simulations of the ligand-free wild-type protein, I836S, I836S phosphorylated, and R841C mutants. This is a representation of small cavities, forming together a whole tunnel. However, if one fragment disappears at some period of MD, the tunnel loses its clearance. From these, Caver Analyst 2.0 calculated the time-dependent volume fluctuations and split the entire tunnel into smaller fragments with a corresponding lifetime. For example, there is a pair of overlapping fragments, colored with purple and green, shown in Figure 4. In general, both are formed with the same set of residues, but have distinct length and width. That is why they belong to separate clusters. The corresponding value in the table shows the frequency of detection during MD. In this case these values could be summed up. Thus, the inner space of WT is large enough to keep the ligand during 20 + 32% of MD simulation time, in the case of I836S—25 + 27%, for R841C—26 + 22%, and only 14 + 15% for the phosphorylated I836S mutant.

## 4. Discussion

In this study, we evaluated the role of I836S amino acid substitution on the ability of mutant AR–LBD to bind DHT ligand using MD simulation. The structural compacting and the gate closure of I836S mutant protein stops the ligand from binding. From these, the subsequent dimerization does not occur because of changes in PPI interface. The obtained results and the proposed protocol enabled our investigation of the effect of mutations on the protein domain induced-fit structural rearrangement and protein–protein interactions by the means of in silico methods.

A simultaneous measurement of the average and maximum radius values, together with a number of channel formations across MD, showed that the entrance bottleneck of the binding site cavity is represented by at least three small tunnels. The number of calculated tunnels mainly depends on H1/H2 helices position along MD. The time of its existence and the radius of each tunnel demonstrate that the entrance conformation of WT AR is more favorable for ligand entering the tunnel. However, we found that a lot depended on the radius of the inner tunnel (Figure 4). For example, the WT AR possesses a cavity, sufficiently extended to fix DHT ligand, and the entrance is formed with a bundle of adjacent small channels. At the same time, the lower probability of getting DHT through the entrance bottleneck due to separation of short tunnels and reduction of its average radius values takes place, as in the case of R841C substitution, previously determined as a variant causing reduced ligand-binding ability. In such a conformation, the number of appropriate frames of MD trajectory becomes smaller when the tunnel’s entrance/gating part is extended. Nevertheless, the maximum radius values observed allow us to suppose that, in general, the process of substrate uptake can take place. Importantly, a similar structural analysis performed for the I836S mutation did not reveal a significant effect on AR–LBD ability to bind ligand, while in a predicted phosphorylated I836S mutant, the time of the gating tunnel and internal cavity existence are significantly shorter. This indicates the structural conditions for disrupted AR protein functionality, including inability to bind ligand, thus inability to form a nuclear homodimer and to bind specific response elements upstream of regulated genes. At the organism level, this would imply development of complete androgen insensitivity syndrome clinical features in all affected family members.

## 5. Conclusions

We conclude that the consequence of I836S amino acid substitution for motif/ligand binding in AR is a structural rearrangement, occurring through the induced-fit mechanism. This mechanism is derived based on our results showing that the diameter of the gateway in the binding site gradually decreases, and then collapses in I836S, R832Q, Y835C, and R841C mutant forms in comparison to the geometry of the WT AR. The phosphorylation of S836 is directly relevant to the described mechanism, because the unmodified residual replacement has no dramatic effect on the binding site shape. Moreover, the analysis of mutant 836 residue phosphorylation is a possible explanation for the issues of homodimerization, since the resulting monomer surface changes indirectly impact the binding site.

Multiple analysis of the MD trajectories showed that LBD itself, represented with a tightly folded globule, but with an AF2 segment, forms a highly ordered hydrophobic surface. The secondary structures of both Apo form and ligand-bound protein alter the flexibility rate in response to a ligand molecule at the active site. Having combined these data with the cavity inspection results and RMSF, we conclude that a significantly higher stabilization of protein folding, caused by mutations, is similar to the effects of ligand binding. Finally, the I836S mutant protein cannot bind the ligand due to compacting and gate closure; therefore, given the changes in PPI interface, the subsequent dimerization does not occur.

## Data Availability

Data available on request due to ethical restrictions. The data presented in this study are available on request from the corresponding author.

## Supplementary Materials

The following are available online at https://www.mdpi.com/article/10.3390/life11070659/s1, Figure S1: Pedigree of patient UKR1901 (IV.4) with identified novel X:67722884 T>G mutation in exon 7 of AR gene and partial electrophoregram of exon 7 of androgen receptor gene sequence, Figure S2: RMSD plot of the protein in the ligand-free and ligand-bound state during 200 ns MD simulations (A). Backbone-based RMSF analysis focused on each individual residue of the protein (B). Conformational space formed based on the clustering of ligand-free (C) and ligand-bound (D) proteins during the last 100 ns of MD simulation, Figure S3: Extended layout of the ligand-induced structural changes related to dimer stability. Dimer structure, colored by chain (cyan and green), with designated time-dependent protein interface (red colored surface) changes for ligand-bound (A) and –unbound AR dimer structures (B), Figure S4 Time-resolved root mean-square fluctuations (RMSF). The amino acids with the highest mobility are shown in red and black; the most stable residues are shown in blue. (A) Time-resolved amino acid RMSFs of AR with and without DHT as a ligand. (B) Time-resolved amino acid RMSFs of AR and I836S. (C) Time-resolved amino acid RMSFs of ligand-bound WT and I836S. (D) Time-resolved amino acid RMSFs of AR and I836S pp mutant, Figure S5 RMSF plot, containing a combined data on ligand-binding along with mutation effect (B), confirming that neither ligand-free mutant I836S protein nor its ligand-bound form demonstrate significant difference from wild-type protein, Figure S6 Evident decrease of the spatial structure stability represented with the Radius of Gyration graphs for each of the studied mutations. A high degree of structural flexibility correlate with the reducing of spikes/arrows amount for DHT-free mutants in comparison to the ligand bound ternary structure, Table S1: The most flexible residues of AR with and without DHT, Table S2: The most flexible residues of AR and I836S, Table S3: The most flexible residues of ligand-bound AR and I836S, Table S4: The most flexible residues of AR and I836S pp mutant.
