# Supplementary material for: Functional Effects In Silico Prediction for Androgen Receptor Ligand-Binding Domain Novel I836S Mutation
Source: Life (Basel). 2021 Jul 6;11(7):659. doi: 10.3390/life11070659 (PMC8303243; doi:10.3390/life11070659)
Supplement: Supplementary file 1 [file life-11-00659-s001.zip › life-1219629-supplementary.pdf]

Functional Effects *in silico* Prediction for Androgen Receptor Ligand-binding Domain Novel I836S Mutation

Alexey Rayevsky, Dmytro Sirokha, Dariia Samofalova, Dmytro Lozhko, Olexandra Gorodna, Inga Prokopenko, Liudmyla Livshits

Supplementary

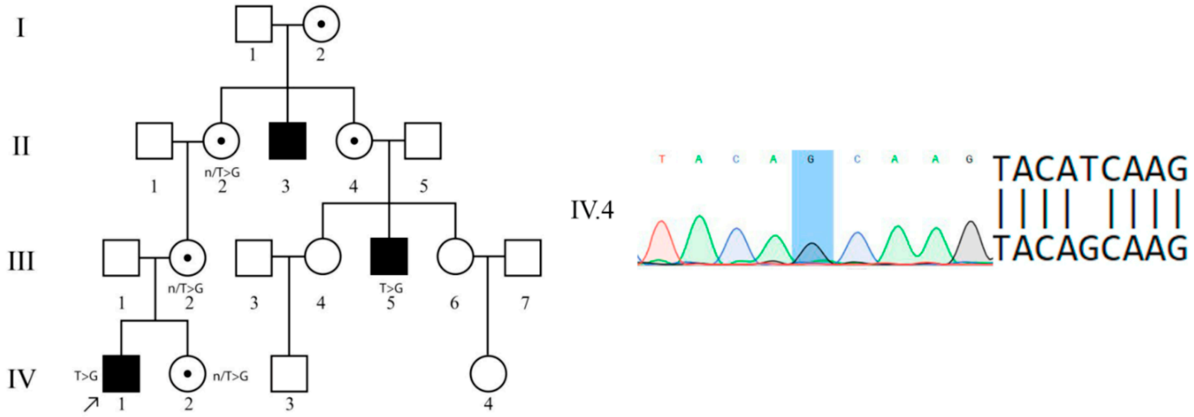

Figure S1. Pedigree of patient UKR1901 (IV.4) with identified novel X:67722884 T>G mutation in exon 7 of AR gene and partial electrophoregram of exon 7 of androgen receptor gene sequence.

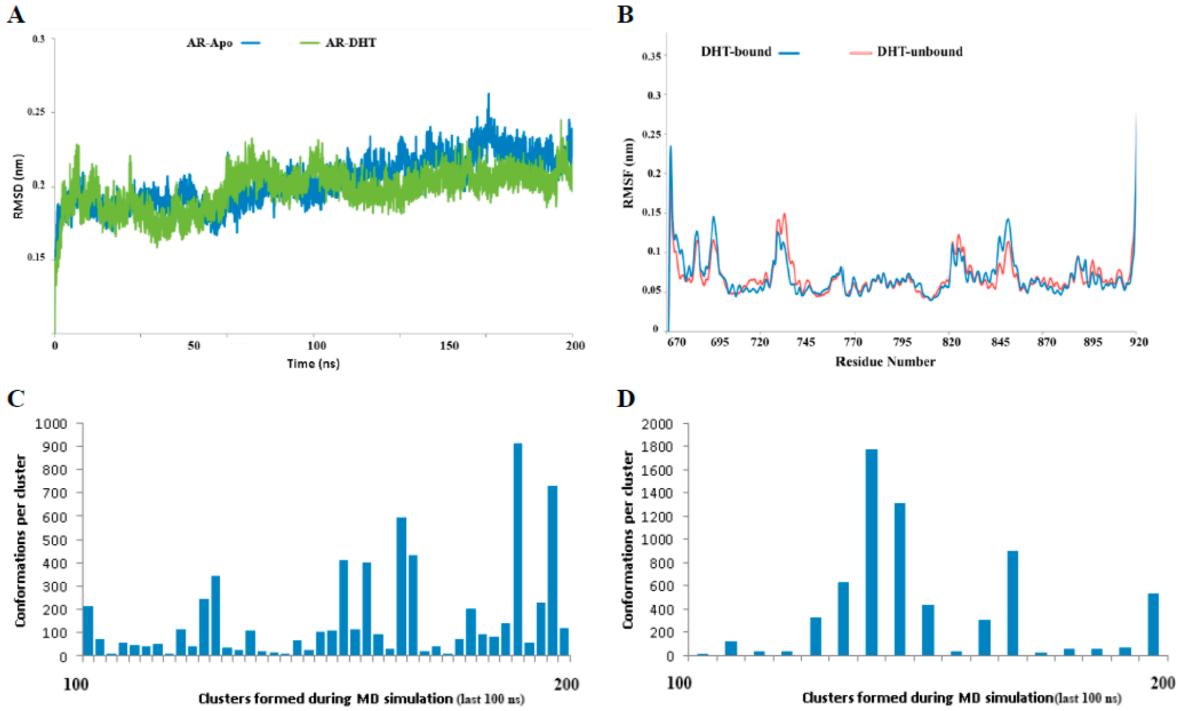

Figure S2. RMSD plot of the protein in the ligand-free and ligand-bound state during 200 ns MD simulations (A). Backbone-based RMSF analysis focused on each individual residue of the protein (B). Conformational space formed based on the clustering of ligand-free (C) and ligand-bound (D) proteins during last 100 ns of MD simulation.

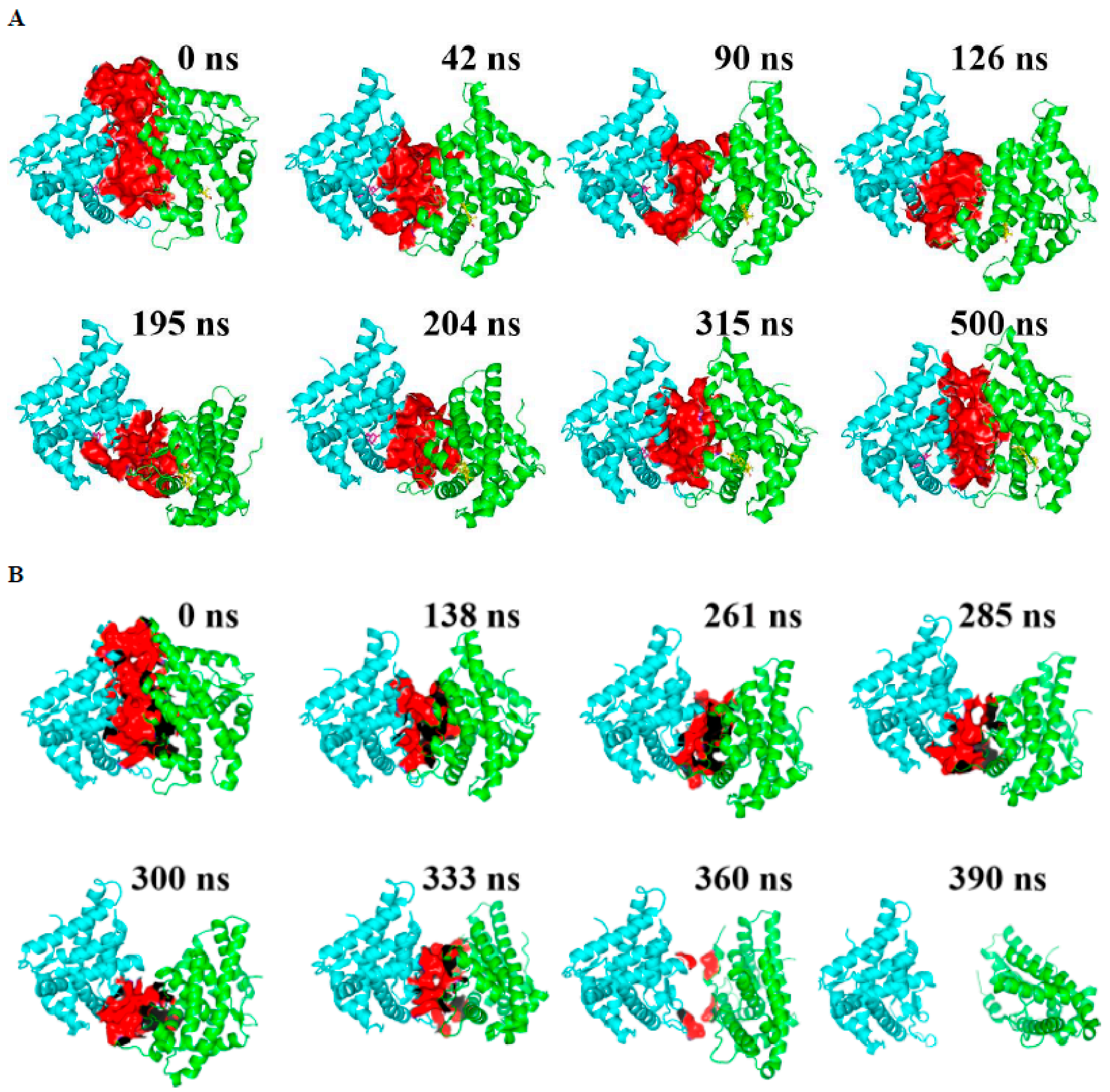

Figure S3. Extended layout of the ligand-induced structural changes related dimer stability. Dimer structure, colored by chain (cyan and green), with designated time-dependent protein interface (red colored surface) changes for ligand-bound (A) and –unbound AR dimer structures (B).

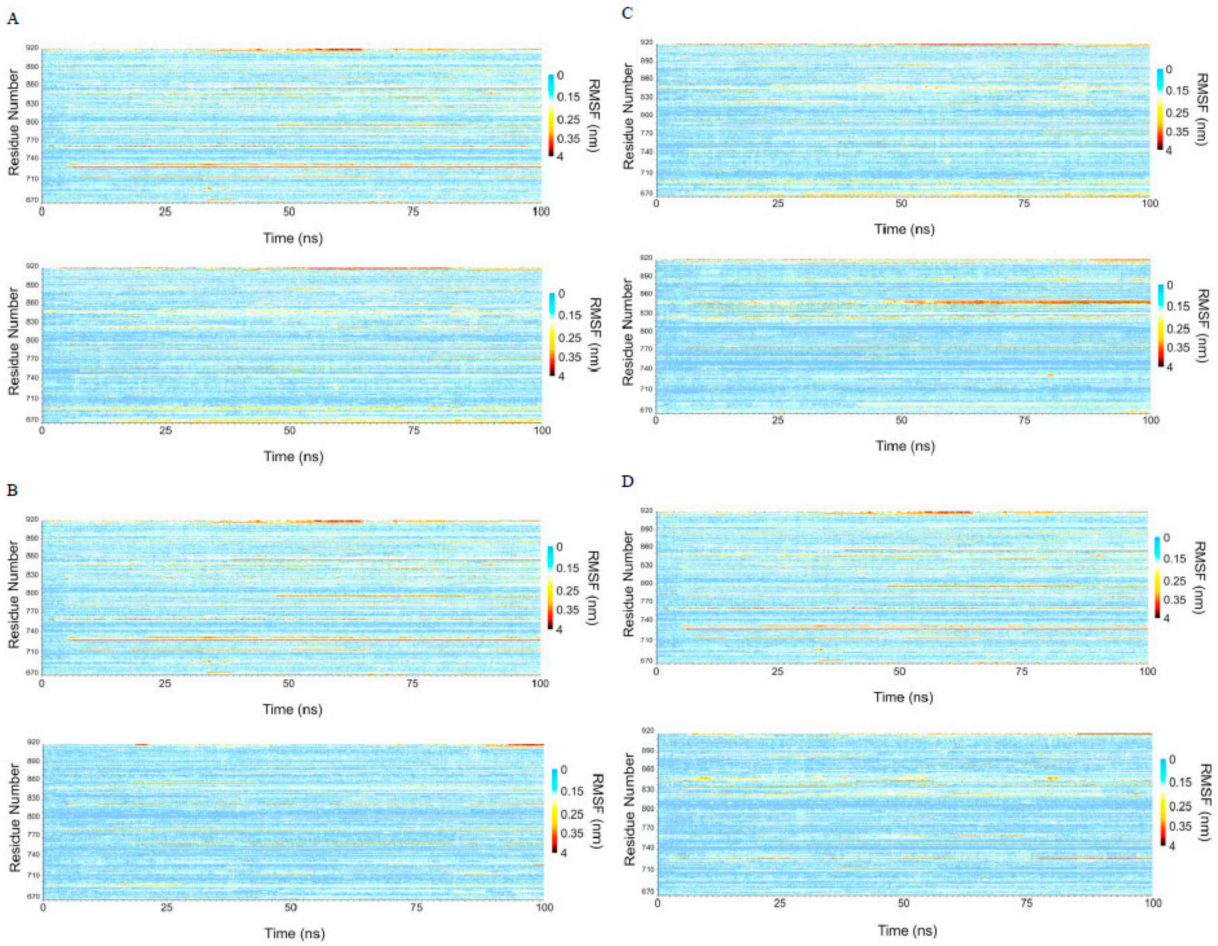

Figure S4. Time-resolved root mean-square fluctuations (RMSF). The amino acids with the highest mobility are shown in red and black; the most stable residues are shown in blue. (A) Time-resolved amino acid RMSFs of AR with and without DHT as ligand. (B)Time-resolved amino acid RMSFs of AR and I836S (C) Time-resolved amino acid RMSFs of ligand-bound WT and I836S. (D) Time-resolved amino acid RMSFs of AR and I836S pp mutant.

Table S1. The most flexible residues of AR with and without DHT.

**AR-Apo**<br>**Most flexible amino acids**	**AR-DHT**<br>**Most flexible amino acids**
712Q	692N
727R	693N
731V	694Q
732D	742W
761R	753R
797W	

Table S2. The most flexible residues of AR and I836S

**AR-Apo**<br>**Most flexible amino acids**	**AR-I836S**<br>**Most flexible amino acids**
711R	695P
727R	780R
728N	803Q
758V	823K

761R	841R
788M	858Y
797W	872R
814F	
856R	

Table S3. The most flexible residues of ligand-bound AR and I836S

**AR-DHT**<br>**Most flexible amino acids**	**AR-DHT –I836S**<br>**Most flexible amino acids**
693N	682E
694Q	683P
695P	684G
742W	774Y
753R	841R
	847R
	848K
	849N
	850R
	851T
	852S

Table S4. The most flexible residues of AR and I836S pp mutant.

**AR-Apo**<br>**Most flexible amino acids**	**AR-I836S**<br>**Most flexible amino acids**
711R	724P
721K	750M
727R	803Q
728N	823K
729L	825Q
730H	837K
731V	841R
758V	850P
761R	855R
788M	872R
797W	886H
813L	
814F	
856R	

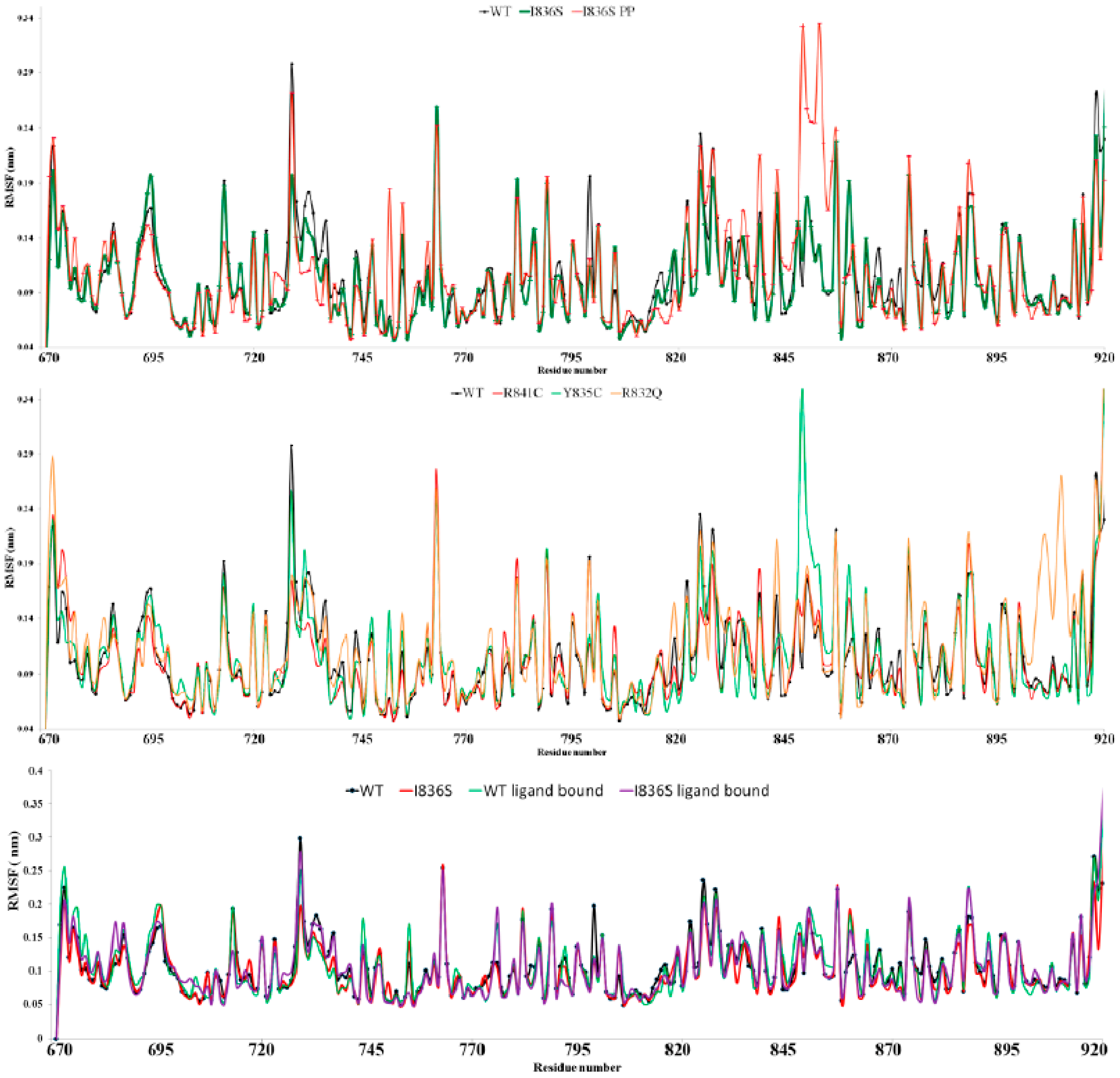

Figure S5. RMSF plot, containing a combined data on ligand-binding along with mutation effect (B), confirming that neither ligand-free mutant I836S protein nor its ligand-bound form demonstrate significant difference from wild-type protein.

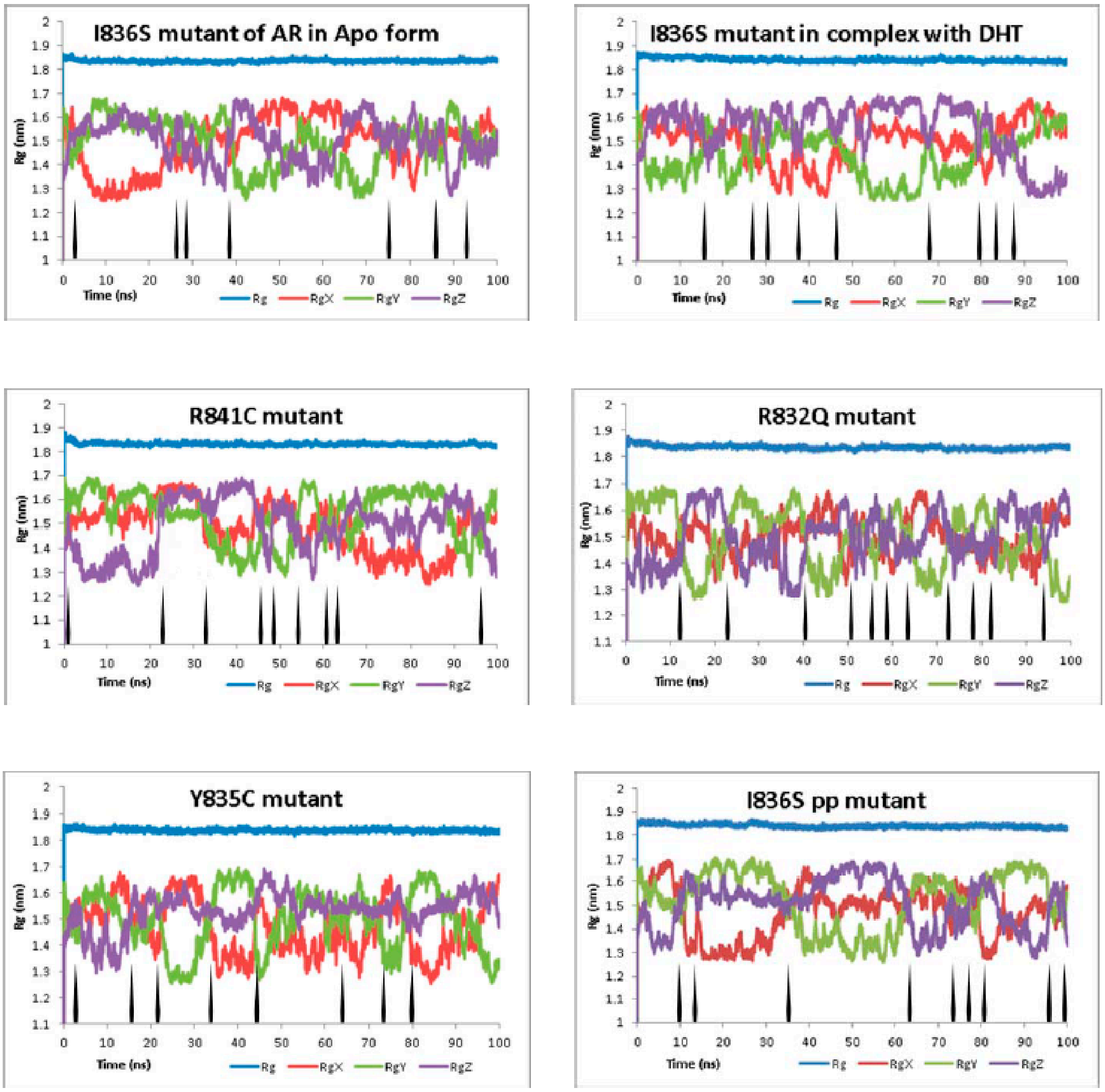

Figure S6. Evident decrease of the spatial structure stability represented with the Radius of Gyration graphs for each of the studied mutations. A high degree of structural flexibility correlate with the reducing of spikes/arrows amount for DHT-free mutants in comparison to the ligand bound ternary structure .
